# Increasing Students’ Long-Term Well-Being by Mandatory Intervention – A Positive Psychology Field Study

**DOI:** 10.3389/fpsyg.2020.553764

**Published:** 2020-10-09

**Authors:** Frida Skarin, Erik Wästlund

**Affiliations:** Department of Social and Psychological Studies, Faculty of Arts and Social Sciences, Service Research Center (CTF), Karlstad University, Karlstad, Sweden

**Keywords:** students, long-term, well-being, change, positive activity intervention, positive psychology

## Abstract

Is it possible to help students experience increased well-being that proceeds by volitional actions from mandatory participation in interventions? The aim of this field study was to better understand the influence of expectancy, motivation, and well-being experiences during a positive activity intervention on long-term behavior change and long-term well-being. The study included 59 students enrolled in a course that included choosing a positive activity that they would plan for and implement in their lives for 6 weeks. The participants answered questionnaires before (pre-measure) and after the intervention (short-term measure), as well as an unannounced follow-up questionnaire 6 months later (long-term measure). Overall, the results indicate the importance of coexisting intrinsic motivation and high expectancy in the outcome and that the key driver of sustained volitional behavior change and experiencing long-term increased well-being is to experience increased well-being during the intervention. The results of the study show that it is possible to help students experience increased well-being that proceeds by volitional actions. The study shows that a mandatory positive activity intervention, including free choice of activity and course of action, can induce new long-term behaviors and long-term increased well-being.

## Introduction

Positive psychology focuses on well-functioning individuals’ mental health improvement and well-being, as compared to the traditional psychology perspective of reducing mental illness (Seligman and Csikszentmihalyi, 2000). It has been argued that the school and university environments are ideal settings to increase and sustain individual well-being (Norrish et al., 2013; Seligman et al., 2009; White and Waters, 2015). The present study was conducted in a university environment and focused on how to use a mandatory positive psychology intervention to increase long-term student well-being. Before deepening the perspective of positive psychology interventions on students, it is useful to define well-being. The most frequently used definitions of well-being available are subjective well-being and psychological well-being (Weiss et al., 2016). Subjective well-being takes a hedonic perspective and has been described as a sophisticated measurement of happiness that focuses not only on circumstances but also on processes and interactions with situations, with other people, and within oneself (Diener et al., 1999). Hedonic aspects of well-being are measured as life satisfaction and a balance between negative and positive affect (Diener, 1984). By contrast, psychological well-being takes a more eudemonic perspective and has been described as self-acceptance, relations with others, autonomy, environmental mastery, purpose in life, and personal growth (Ryff, 1989). Eudemonic aspects of well-being comprise a broader and multifaceted set of needs for optimal functioning, personal growth, self-determination, and having a meaningful life. The terms *subjective well-being* and *happiness* are often used interchangeably (Lyubomirsky, 2001; Diener et al., 1999), so we have used them interchangeably in this paper.

From the positive psychology perspective, individuals can make the most of their resources by using their personality traits and signature strengths in ways that cultivate their specific capacities and increase well-being. This approach could be appealing and easily accessed by students. Several meta-analyses (see, for instance, Bolier et al., 2013; Hone et al., 2015; Sin and Lyubomirsky, 2009; Weiss et al., 2016) have enforced the efficacy of positive psychology interventions in increasing well-being and ameliorating depressive symptoms, especially for those in the middle range of the well-being continuum. Lyubomirsky (2008) presented 12 positive activity interventions, which can be adjusted to suit each individual. The positive activities have been shown to increase well-being if implemented in everyday life for 8 weeks. Lyubomirsky argued that when mindful practical actions are executed in everyday life, positive emotions emerge connected to the area in focus, which reproduce themselves (Lyubomirsky, 2008). Table 1 provides a brief presentation of the nature of the activities and how they can be practiced.

**TABLE 1:** Lyubomirsky’s 12 positive activities with examples of behaviors that can be implemented in everyday life.

Positive activity	Description examples
Expressing gratitude	Observe, appreciate, and write down good things in your life
Cultivating optimism	Notice and express positive sides of things
Avoiding over-thinking and social comparison	Limit rumination to one “rumination hour” per day
Practicing acts of kindness	Offer help, give (honest) compliments
Nurturing social relationships	Express love, be supportive, show appreciation
Developing strategies for coping	Find meaning in traumas, turn to social support
Learning to forgive	Write forgiveness letter(s)
Increasing flow experience	Engage in activities with balance between challenge and control
Savoring life’s joys	Be mindful when doing more of things you enjoy
Committing to your goals	Learn and practice authentic, harmonious goals
Practicing religion and spirituality	Perform spiritual activities, pray
Taking care of your body and soul	Practice meditation or physical activity

The very nature of an intervention implies a targeted action during a limited timeframe with the aim of changing a specific behavior or state of affairs. Thus, the goal of the intervention is to help individuals adopt new behaviors such as committing to goals. Understandably, behavior change interventions are carried out with the intention of changing behaviors, not only during the intervention itself but also having sufficient impact to sustain the intervention-induced behavior. Therefore, it is necessary to investigate the effects of behavior change interventions, in terms of both short-term effects in immediate conjecture with the intervention and long-term effects in terms of sustained behavior.

### Motivation

When participating in a behavior change intervention, motivation is needed to carry through with it. Thus, to understand the present study’s angle on students’ motivation, we briefly present the related motivational theory. As Sheldon and Ryan (2011) argued, Self-Determination Theory (SDT) (Deci and Ryan, 2008) is a motivational theory meeting positive psychology, both in its framework for understanding optimal functioning and in the need for autonomy and met needs for succeeding in well-being interventions. According to SDT, three basic human needs that activate behavior are central for motivation to occur: competence, autonomy, and relatedness. The desire to control the outcome of one’s actions (competence); the need to act in harmony with true interest and values and out of free will (autonomy); and the need to perform actions that include belongingness, social support, and togetherness (relatedness) are associated with psychological health and effective performance. SDT divides motivation into intrinsic and extrinsic forms. Intrinsic motivation is the drive to do things autonomously, out of curiosity, for the joy and internal reward of feeling good, excited, or pleased. Intrinsic motivation reflects the instant gratification of performing a behavior. Extrinsic motivation is a controlling form of motivation and typically refers to the drive to do things in order to receive an external reward, such as money, praise, or awards, or to avoid punishment, such as scolding or disgrace (Deci and Ryan, 2008). In SDT, the latter form of extrinsic motivation is referred to as external regulation. Ryan (2012) explained that extrinsic motivation can be internalized, where introjection is a controlled (and therefore the least autonomous) form of extrinsic motivation. Examples include parents reacting to their child’s academic performance by rewards or punishment, which leads to negative well-being consequences for the child because of contingencies of self-worth and usually does not result in long-term performance because of the non-volitional nature. A more autonomous form of internalization is identified regulation; for example, the child in the above example would understand and accept the value of the behavior (doing well at school). The most autonomous form of internalization is integrated regulation, which refers to when the behavior has been adopted and becomes in line with the individual’s own identification and needs. Thus, externally motivated behavior can become volitional. Extrinsically motivated behaviors normally do not transform into intrinsic ones because of their instrumental focuses (Ryan, 2012). However, different types of motivation can coexist, and a behavior can start in one form and transform to another. The difference between intrinsic motivation and a fully integrated extrinsic motivation – that is, integrated regulation – is that the former is, by definition, doing something for the joy and interest of it, while the latter is doing something for the value and meaning of it.

Lyubomirsky developed a self-diagnostic test – the Person–Activity Fit Diagnostic – which is recommended to be taken before choosing a positive activity to engage in, to determine which activity would be most valuable for each individual. The Person–Activity Fit Diagnostic consists of 12 activities to consider regarding five reasons one would possibly engage in them: how *natural* it would feel, how much one would *enjoy* doing it, how much *value* and identification it would bring, how *guilty* one would feel to not do it, and how one would do it because the *situation* or someone else forces them to do it. The reasons for engaging in positive activities tap into the different kinds of motivation: *natural* and *enjoy* tap intrinsic motivation, *value* taps identified motivation, *guilty* taps introjected motivation, and *situation* taps external motivation (Lyubomirsky, 2008). However, even with prerequisites for motivation in place, behavior change is still subject to another factor: the individual’s beliefs (Bandura, 1997). The belief of what the future will bring is called expectation.

### Expectation

Our expectations seem to influence what the future actually brings. Murphy (1999) researched the influence of expectancy on the outcome of therapy and defined expectancy as “the client’s hope and expectancy of change as a result of participating in therapy.” From an intervention perspective, the idea of expectations having significant effects on outcomes of the intervention is intriguing. Previous research shows that expectation seems to be as important as the activity itself (Volkow et al., 2003).

Bandura (1977) self-efficacy theory focused on expectancies for success and divided expectancy into self-expectancy and outcome expectancy. Self-expectancy represents individuals’ belief in their ability to perform a certain behavior, whereas outcome expectancy represents the belief that a certain behavior will lead to a certain outcome. Rather than focusing on different types of expectancy, the present study uses the term *expectancy* to describe the participants’ general expectation of the overall possible success rate that their participation in the intervention may bring.

Behavior change interventions are carried out with the intention of changing specific behaviors; however, the ultimate goal is usually not the target behavior itself but the outcome – the experienced gain – which makes change worthwhile for the individual. Therefore, to understand well-being interventions, we must differentiate between the changed behavior and its outcome.

### Outcome and Well-Being of Students

A desired outcome from well-being interventions in a school setting is that the students should experience increased well-being. The very nature of an intervention implies a targeted action during a limited timeframe with the aim of changing a specific behavior or state of affairs. Thus, the goal of this intervention was to help students adopt a new behavior and increase their well-being. Understandably, interventions are enacted with the intention of changing behaviors, with enough impact to sustain the behavior. Consequently, a study of the effects of interventions must include short-term and long-term effects in terms of sustained behavior and sustained well-being.

It is helpful to promote a path to better mental health and well-being in an educational setting where students easily can access the perspective and be guided on how to apply and implement it in everyday life to build a foundation for the rest of their lives. A lot of previous research has examined well-being interventions in a school setting with a set course of action for students to follow (see, for instance, Shoshani and Steinmetz, 2014; Lambert et al., 2019). However, personal fit with positive activities has been argued to be important (Lyubomirsky, 2008) and could save monetary, personnel, and time resources. Additionally, from a motivational perspective, it would be desirable to make an externally prompted behavior become intrinsically or fully externally integratedly motivated. Therefore, the present study was conducted with the distinguishing parameters of mandatory participation in the intervention, but where the participants were free to choose one positive activity themselves and plan the implementation of the activity to fit their everyday lives. Research is needed in this course to create interventions that help students experience long-term increased well-being. Hence, the aim of present field study was to investigate the following research questions in the light of an externally prompted positive activity intervention:

RQ1: How does expectancy influence the effect of motivation on experienced well-being change?
RQ2: How do students’ motivation and expectancy relate to long-term behavior change and experienced long-term well-being increase?

## Materials and Methods

**FIGURE 1:**
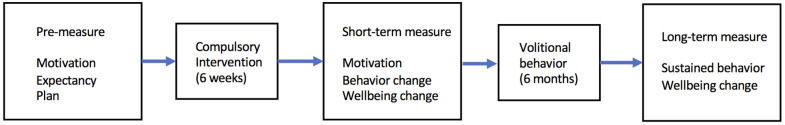
Study timeline.

The study was based on a 6-week well-being intervention. Participants were given questionnaires before and after the intervention, as well as an unannounced follow-up measurement 6 months later (see Figure 1). Although the intervention was a compulsory class assignment, participation in the study was voluntary, and nothing was said about follow-up measurements. The participants received no support to continue the intervention-induced behaviors after the intervention. Therefore, results from the follow-up measurements can be taken to indicate the participants’ volitional sustained behavior. Considering the motivational processes of the SDT, the participants were asked to specify whether their motivation was attributed to the ambition of being a good student and receiving good grades (encapsulated in extrinsic motivation in the present study) or to an inner will and genuine interest (intrinsic motivation) in behavior change.

### Participants and Recruitment

As part of a mandatory course in the first year of the psychology program at Karlstad University, Sweden, students were obliged to study the 12 evidence-based positive activity interventions and their implementation (Lyubomirsky, 2008) and select one that they would implement in their lives for the following 6 weeks. The assignment included planning for implementation that would fit the students’ lives and writing a paper about their experiences linked to the literature. The students’ plans were written down individually and included their choice of activity, activity frequency, time and place for the activity, as well as time, place, and form for journaling the activity. Among the students from three different cohorts of the course, volunteers were recruited for the present study and required to answer questionnaires before and after the intervention. In addition, participants were approached 6 months after the intervention ended to answer an unannounced follow-up questionnaire. The questionnaires were handed out in printed form: the pre-measure before the first lecture of the course, the short-term measure after the last lecture of the course, and the long-term measure before a lecture in a different course 6 months later. At the pre-measure, the students were asked about their plan (positive activity choice, frequency, and course of action), motivation (intrinsic versus extrinsic), and expectations (high versus low) regarding the intervention results. At the short-term measure, the students were asked about their experienced well-being change (if any), their behavior change, and their motivation at this point. At the post-measure, the students were asked about their experienced well-being change and about their current engagement in the target behavior. For an illustrative study overview, see Figure 1.

The handouts resulted in 59 complete questionnaires (with negligible drop-out). The sample consisted of 26 percent male and 74 percent female participants, ranging in age from 19 to 44 years old, with a mean age of 24.8 (SD = 5.93). Each questionnaire took approximately 15–20 min to complete. To reveal spontaneous volitional behavior change motivated from within the participants themselves, the follow-up measurement was not announced beforehand.

### Material

Well-being can be measured by a single-item scale that simply asks people how happy they are (Abdel-Khalek, 2006) or by standardized batteries of questions. Lyubomirsky (2008) study on positive activity interventions applied the latter, specifically the Subjective Happiness Scale or SHS (Lyubomirsky and Lepper, 1999). The SHS is a global, subjective measurement of happiness that includes absolute ratings as well as comparison to other people. Lyubomirsky and colleagues argued that positive activities increased happiness, based on before and after measurements using the SHS. In line with other studies (Gardner et al., 1998; Hoeppner et al., 2011), the present study used a single-item scale to measure happiness before and after the intervention, as well as at the 6-month follow-up.

#### Pre-measures

The questionnaires containing the pre-measures were distributed before the intervention began. In addition to basic demographics, the participants were asked questions regarding their belief in the intervention (expectancy) and how often they intended to execute the new behavior (plan). Expectancy is most often measured by a single-item scale (e.g., Ilgen et al., 1981; Wanous et al., 1997). Therefore, we used a seven-point Likert scale that asked the participants to indicate how much happier they expected to be after carrying out the intervention, with 1 being “not at all happier” and 7 being “a lot happier.” Participants’ new behavior plan was measured with the question “How often do you plan to carry out your chosen behavior during the course?” The response choices were “never,” “very occasionally,” “once per month,” “several times per month,” “once per week,” and “several times per week.”

#### Short-Term Measurement

The questionnaires measuring the short-term results were distributed ahead of a lecture at the end of the course and contained questions regarding motivation (intrinsic versus extrinsic), how often the new behavior was executed (short-term behavior change), and how much happier the participants felt after the intervention compared with before (experienced short-term change in well-being). In line with Markland and Hardy (1997), motivation was measured with a seven-point Likert scale indicating the level of motivation participants had to carry through the intervention, ranging from 1 (“not at all/doing it just because of the course;” that is, extrinsically motivated) to 7 (“very motivated/could do it totally voluntary;” that is, intrinsically motivated). Short-term behavior change was measured by self-report: “How often did you carry out the behavior you had chosen during the course?” with the responses “never,” “very occasionally,” “once a month,” “several times a month,” “once a week,” and “several times a week.” Given that our intention was to measure change rather than objective happiness, we used the self-report question “How much happier are you now compared to before the intervention?” to measure the participants’ experienced short-term change in well-being, if any; responses ranged from 1 (“not at all”) to 7 (“very much”). Self-reported measurements for well-being (Armenta et al., 2014) and behavior change (Cohn and Fredrickson, 2010) align with previous research.

#### Long-Term Measurement

The questionnaires measuring long-term results were distributed 6 months after the end of the course. Specifically, participants were asked a single-item question to measure long-term behavior change: “How often have you carried out the behavior you had chosen during the course since the course ended?” The response choices were “never,” “very occasionally,” “once a month,” “several times a month,” “once a week,” and “several times a week.” To measure the participants’ experienced long-term change in well-being, if any, they were asked to answer the question “How much happier are you now compared to before the intervention?” using a seven-point scale.

## Results

**FIGURE 2:**
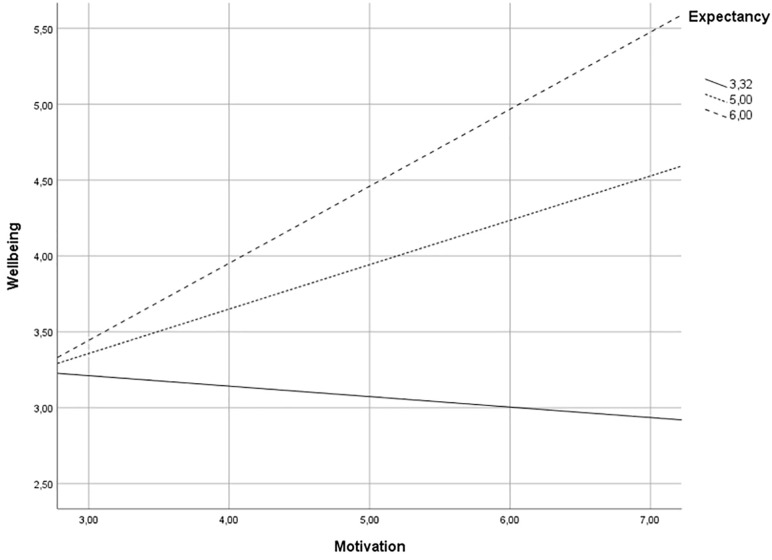
The conditional effect of the focal predictor motivation (extrinsic to intrinsic) on the wellbeing (experienced change) at different levels of expectancy.

In answering RQ1, we first looked at the reported behavior change. All participants set ambitious plans, including significant behavior changes during the intervention and reported altered behavior. Ninety-seven percent performed their new behavior one or several times per week. Experienced short-term well-being change indicated an increase (responses of 5, 6, or 7 on a seven-point scale) for 40.6 percent of the participants. To investigate the effect of motivation and expectancy on experienced well-being change, we conducted a simple moderation analysis using the model 1 PROCESS procedure for SPSS (Hayes, 2013), which indicated a moderating effect of expectancy on the relationship between motivation and experienced well-being change. The moderation analysis indicated that the overall model was significant (*p* = 0.0032, *R*^2^ = 0.25). A follow-up spotlight analysis at the 16th, 50th, and 84th percentiles indicated that the positive effect of motivation on experienced well-being change was significant only when expectancy was high (see Figure 2).

As Figure 2 shows, the more intrinsically motivated the participant, the greater the increase in experienced well-being change following the intervention, provided that the participant had a strong belief that the intervention would lead to a desired outcome. Consequently, without high expectancy, intrinsic motivation did not have a significant effect on experienced well-being change.

To answer RQ2, we utilized an unannounced follow-up questionnaire 6 months after the completion of the intervention finished, in which participants were asked about their spontaneous, self-initiated sustained behavior and its outcome. We were interested in any differences between those who spontaneously continued the intervention-induced behavior and those who did not. For a fairer picture, we divided the participants into two groups based on how frequently they reported engaging in the intervention-induced behavior. These two groups were (1) those who changed behavior (that is, engaged in intervention-induced behavior once a week or more: *n* = 12, 20.33 percent of the original sample) and (2) those who did not change behavior (that is, never engaged in intervention-induced behavior: *n* = 13, 22.03 percent of the original sample). Reports falling in between *change* and *not change* (that is, those who engaged in intervention-induced behavior “occasionally” or “one or a few times per month”) were classified as neither-nor and were excluded from the analysis.

Independent sample *t*-tests were run between those who had changed their behavior and those who had not. The *t*-test results indicated that the participants in group 1 (with significantly changed behavior) reported higher levels of expectancy [*t*(20) = 2.936, *p* = 0.009], measurement of experienced short-term change in well-being [*t*(21) = 2.478, *p* = 0.022], and measurement of experienced long-term change in well-being [*t*(23) = 2.310, *p* = 0.03]. Surprisingly, there was no significant difference in motivation between the two groups (see Table 2 for means and standard deviations).

**TABLE 2:** Descriptive for participants who changed vs. did not change behavior (group 0 = did not change and 1 = changed behavior) * = *p* < 0.05.

	Group	N	M	SD
Motivation	0	12	4.50	1.57
	1	11	5.45	1.44
Expectancy*	0	11	4.18	0.60
	1	11	5.09	0.83
Experienced short-term wellbeing change*	0	12	3.42	1.51
	1	11	4.82	1.17
Experienced long-term wellbeing change*	0	13	4.08	1.12
	1	12	5.00	0.85

Lastly, in order to compare the relationship between short-term change in well-being, long-term change in well-being, and sustained volitional behavior change, we conducted a correlation analysis. The results show a slightly higher positive correlation between short-term change in well-being and sustained volitional behavior change (see Table 3).

**TABLE 3:** Correlations between experienced short-term change in wellbeing and the following variables: long-term change in wellbeing and sustained volitional behavior change (group 1 and 2).

		Short-term wellbeing change	Long-term wellbeing change	Long-term behavior change
Short-term wellbeing change	Pearson	1	0.483*	0.476*
	Sig. (2-tailed)		0.020	0.022

Long-term wellbeing change	N	23	23	23
	Pearson	0.483*	1	0.434*

Long-term behavior change	Sig. (2-tailed)	0.020		0.030
	N	23	25	25
	Pearson	0.476*	0.434*	1
	Sig. (2-tailed)	0.022	0.030	
	N	23	25	25

*Correlation is significant at the 0.05 level (two-tailed).

## Discussion

The aims of this field study were to investigate (1) how expectancy influences the effect of motivation on experienced well-being change as a result of an externally prompted intervention and (2) how motivation and expectancy relate to volitional long-term change in behavior and experienced well-being. Overall, the results indicate the importance of coexisting intrinsic motivation and high expectancy in the outcome and that the key driver of sustained volitional behavior change and experiencing long-term increased well-being is to experience increased well-being during the intervention.

In line with previous research (e.g., Deci and Ryan, 2000; Murphy, 1999; Strecher et al., 1986), the present study indicates that crucial factors for successfully changing behavior are intrinsic motivation and the participant’s expectancy that the intervention will lead to desired outcomes. However, the positive relationship between intrinsic motivation and intervention outcome (represented in this study by experienced well-being change) hinges on expectancy. Thus, neither intrinsic motivation nor expectancy alone seems to lead to a positive outcome; the two factors must be present simultaneously.

As graduate psychology students, participants in this study were most likely to have gained both intrinsic motivation and belief in the intervention from studying previous research (lectures, mandatory literature, and other available literature and information sources) about intervention activities. Therefore, we propose that the use of information and study materials may be an efficient way for intervention designers to help participants increase their intrinsic motivation and belief in the intervention. However, we should note that the participants’ levels of expectancy were measured at the beginning of the course, which meant they were not a result of class activities.

In line with Bandura (1977) and Deci and Ryan (2000), the present study indicates that intrinsic motivation and expectancy are important, for both changing behavior and experiencing well-being during the intervention. Before the start of the study’s intervention, all participants seemed to have high intrinsic motivation and ambition (that is, they set high goals) to engage in target behaviors. During the intervention, they all also reported engagement in the new behaviors in line with these high standards. When the intervention ended, the participants did not have any obligation or external encouragement to continue any behavior. Nevertheless, more than one-fifth of them chose to continue the target behavior and were still engaged in it 6 months later. Thus, the externally prompted behavior had turned into a volitional behavior.

To tease out the drivers of this transformation, we can compare participants who sustained their target behavior with those who quit as soon as the intervention ended. The results indicate that both expectancy and experienced well-being change differed between the two groups. Participants who continued their target behavior reported higher levels of expectancy before the intervention began and higher levels of experienced well-being change as a result of the intervention. Interestingly, the two groups showed no difference in reported intrinsic motivation. This finding indicates that actual experience of the intervention is a stronger predictor than self-reported motivation.

Thus, an externally prompted intervention such as a mandatory course can help students to volitionally make changes in their everyday behavior, even long-term, which can increase their well-being. As Lyubomirsky et al. (2011) discussed, results vary from studies with differing recruitment methods, such as voluntary versus non-voluntary selection. The results of studies in which participants voluntarily participated in interventions, with full awareness of the desired outcome (such as to improve well-being), tend to be more successful than the results of studies in which participants were unaware of the purpose of the study, such as participating in exchange for credits after being told that the study was a “cognitive exercise” (cf. Seligman et al., 2005; Fordyce, 1977; with Lyubomirsky et al., 2005; Sheldon and Lyubomirsky, 2006). Similar to the suggestion that knowledge about the intervention’s purpose may moderate the intervention’s effectiveness (Lyubomirsky et al., 2011), the present study included participants who were aware of the intervention’s purpose, although they did not self-select to participate. However, the effectiveness might rely on the motivation rather than on self-selection itself; this is shown in the present study in terms of the influence of intrinsic motivation on long-term behavior change and experiencing well-being change and also in terms of an extrinsic motivational transformation process. Consequently, in line with Deci and Ryan (2000), the results of the present study imply that the initial external motivation, in which behavior was controlled by the mandatory course setting, seemed to transform during the intervention and become integrated. Hence, integrated regulation of external motivation led to self-determined extrinsic motivation – the most complete internalization of extrinsic motivation (Deci and Ryan, 2000). In sum, the present study highlights the long-term impact of a 6-week mandatory positive activity intervention in school, which was shown to help students experience increased well-being.

### Limitations and Future Research

Knowledge can influence expectancy and motivation. Therefore, a limitation of this study is that the participants had studied research and learned more about the intervention activities during the course and had therefore gained knowledge that has not been measured before or after the intervention. Hence, we cannot be certain about the reasons for increased motivation and expectancy during the intervention. Accordingly, future research is needed on how to help students raise their intrinsic motivation, transforming external motivation into integrated self-regulation, and on how studying research may influence motivation and expectancy. A possible limitation of this study is the issue of self-selection, as the sample consisted of students who had voluntarily enrolled in the psychology program. Nevertheless, the results indicated differences in motivation among the participants, and those who were more intrinsically motivated were more successful at changing behavior and reaching a higher outcome from the intervention. Our choice of measuring change of well-being rather than choosing an objective measurement of well-being is a limitation of the study; we have tried to eliminate the negative effects of this limitation through a clear and replicable study and report. The reason for our decision to measure change rather than objective happiness was that we wanted to capture the participants’ experience of change with the aim of presenting a deeper layer including reflection of a wider time perspective rather than a more minutes-based “right-now” happiness. We argue that asking about change encourages participants to reflect on their experienced change rather than report a happiness rate that applies to a shorter timeframe. Another way to do this would have been to collect objective measurement of happiness, so we consider this choice to also be a limitation.

## Conclusion

The results of this study indicate that predictors of long-term behavior change through a positive activity intervention are the participants’ expectancy before the intervention and their experience of desired outcome (that is, enhanced well-being) during the intervention. Those participants who continued to engage in intervention-induced behavior of their own volition 6 months after the intervention experienced a long-term increase in well-being. Intrinsic motivation modified the participants’ effect of expectancy and their experience of the desired outcome during the intervention. Accordingly, those who were intrinsically motivated and believed in the intervention experienced more change in well-being, which motivated them to proceed with the behavior that had increased their well-being. These results add to the knowledge regarding the relationships among motivation, expectancy, and well-being outcome and, consequently, how experienced short-term change in well-being can predict long-term behavior change and experienced long-term change in well-being. By strengthening prerequisites for intrinsic and fully integrated extrinsic motivation, belief in the intervention, as well as making positive outcomes of the intervention explicit, students can be supported to make volitional long-term behavior changes.

## Data Availability Statement

The raw data supporting the conclusions of this article will be made available by the authors, without undue reservation.

## Ethics Statement

Ethical review and approval was not required for the study on human participants in accordance with the local legislation and institutional requirements. The patients/participants provided their written informed consent to participate in this study.

## Author Contributions

FS contributed with the initial idea generation, data collection, data analysis, and manuscript writing. EW contributed to supervision, study design improvement, interpretation of the results, and critical reviews of the manuscript. Both authors contributed to the article and approved the submitted version.

## Conflict of Interest

The authors declare that the research was conducted in the absence of any commercial or financial relationships that could be construed as a potential conflict of interest.
